# TSPO Ligands Protect against Neuronal Damage Mediated by LPS-Induced BV-2 Microglia Activation

**DOI:** 10.1155/2022/5896699

**Published:** 2022-03-30

**Authors:** Baoyu Ma, Yongxin Liu, Xiao Zhang, Rui Zhang, Zhenjiang Zhang, Zhihao Zhang, Jie Liu, Zhaodong Juan, Xiaotong Sun, Lina Sun, Jingyao Huang, Jinpeng Feng

**Affiliations:** ^1^Shandong Provincial Medicine and Health Key Laboratory of Clinical Anesthesia, School of Anesthesiology, Weifang Medical University, Weifang 261053, China; ^2^Department of Thoracic Surgery, Weifang People's Hospital, Weifang 261000, China

## Abstract

Neuroinflammation is a critical pathological process of neurodegenerative diseases, and alleviating the inflammatory response caused by abnormally activated microglia might be valuable for treatment. The 18 kDa translocator protein (TSPO), a biomarker of neuroinflammation, is significantly elevated in activated microglia. However, the role of TSPO in microglia activation has not been well demonstrated. In this study, we evaluated the role of TSPO and its ligands PK11195 and Midazolam in LPS-activated BV-2 microglia cells involving mitophagy process and the nucleotide-binding domain-like receptor protein 3 (NLRP3) inflammasome activation. In the microglia-neuron coculture system, the neurotoxicity induced by LPS-activated microglia and the neuroprotective effects of PK11195 and Midazolam were evaluated. Our results showed that after being stimulated by LPS, the expression of TSPO was increased, and the process of mitophagy was inhibited in BV-2 microglia cells. Inhibition of mitophagy was reversed by pretreatment with PK11195 and Midazolam. And the NLRP3 inflammasome was increased in LPS-activated BV-2 microglia cells in the microglia-neuron coculture system; pretreatment with PK11195 and Midazolam limited this undesirable situation. Lastly, PK11195 and Midazolam improved the cell viability and reduced apoptosis of neuronal cells in the microglia-neuron coculture system. Taken together, TSPO ligands PK11195 and Midazolam showed neuroprotective effects by reducing the inflammatory response of LPS-activated microglia, which may be related to the enhancement of mitophagy and the inhibition of NLRP3 inflammasome.

## 1. Introduction

The main characteristic of neurodegenerative diseases is chronic progressive loss of neurons in the brain and spinal cord. The pathophysiological processes of neurodegenerative diseases remain unclear. In the past, relevant researches are mainly focused on neuroinflammation [1, 2], abnormal protein conformational properties [3], blood-brain barrier structure, and functional integrity disorder [4]. In recent years, the homeostasis regulation on mitophagy and neuroinflammation of microglia cells have become the hotspots in the field [5–8].

Microglia are a type of tissue-specific macrophages presented in the central nervous system (CNS) and possess an immune surveillance function [9]. Studies have shown the involvement of microglia activation in neuroinflammation and neurodegenerative diseases, such as Alzheimer's diseases (AD), Parkinson's diseases (PD), and amyotrophic lateral sclerosis (ALS). [10, 11] As a biomarker of microglia activation and neuroinflammation [12], the translocator protein (TSPO) has attracted a lot of attention [13–15]. The TSPO expression is increased in neurodegenerative diseases suggesting that TSPO is involved in the development and progression of neurodegenerative diseases [16, 17]. And mitophagy is inextricably linked to microglia activation and neuroinflammation [5–8]. Therefore, it is possible that TSPO affects neurodegenerative diseases by regulating mitochondrial autophagy. Decreased efficiency of mitophagy caused by the increase of TSPO in activated microglia [18, 19] may lead to the accumulation of reactive oxygen species (ROS) that could activate NLRP3 inflammasome to induce inflammatory response [20]. Activated microglia can stimulate astrocytes and recruit peripheral macrophages into the brain. Under normal circumstances, microglia as a housekeeper promotes neuronal well-being and normal operation. However, abnormally activated microglia also have the capacity to damage and kill neurons. Therefore, the intervention of neuroinflammation caused by abnormally activated microglia is a promising approach for the prevention and treatment of neurodegenerative diseases.

Our previous studies have shown that TSPO ligands PK11195 and Midazolam have potential therapeutic effects on neuroinflammation [21]. In the current study, we investigate the antineuroinflammatory effects and possible mechanisms of PK11195 and Midazolam in LPS-activated BV-2 microglia cells *in vitro*. The involvement of mitophagy in this process was examined. Finally, the protective effects of PK11195 and Midazolam against neuroinflammation-induced neurotoxicity in NSC34 and HT-22 neuronal cells cocultured with BV-2 microglia were explored.

## 2. Materials and Methods

### 2.1. Cell Culture

The BV-2 mouse microglial cell line was purchased from the Cobioer Biological Technology Company (Nanjing, China). The NSC34 mouse neuronal cell line was purchased from the Wuhan Fine Biotech Co., Ltd. (Wuhan, China). The HT-22 mouse hippocampal cell line was purchased from the Procell Life Science&Technology Co., Ltd. (Wuhan, China). BV-2 microglia cells were cultured in 1,640 medium; NSC34 and HT-22 cells were cultured in DMEM, with 10% fetal bovine serum (Gibco, Shanghai, China).

### 2.2. BV-2 Microglia Treatment

The BV-2 microglia cells were randomly divided into six groups including a control group, PK11195 (0.5 *μ*M, Sigma, Aldrich, USA) group, LPS (1 *μ*g/mL, Solarbio, Beijing, China) group, PK11195+LPS group, Midazolam (15 *μ*M, NHWA, Jiangsu, China) group, and Midazolam+LPS group. In both the PK11195+LPS group and Midazolam+LPS group, cells were pretreated with PK11195 (0.5 *μ*M) or Midazolam (15 *μ*M) for 1 h before treating with LPS for 6 h, and all the cells were harvested after treatment (Figure S1).

### 2.3. BV-2 Microglia and NSC34 Neuronal Cell Coculture System

NSC34 neuronal cells were cocultured with conditioned medium (CM) from BV-2 microglia cells or were cocultured with BV-2 microglia cells in Transwell (24 mm Transwell with 0.4 *μ*m pore size insert; Corning, New York, USA). For CM coculture system (Figure S2 A), the cells were randomly divided into four groups: control, LPS, PK11195+LPS, and Midazolam+LPS. In the LPS group, BV-2 microglia were activated by 10 ng/mL LPS. In the last two groups, BV-2 microglia cells were pretreated with PK11195 (0.5 *μ*M) or Midazolam (15 *μ*M) for 1 h followed by LPS treatment for 6 h; then, the BV-2 microglia cells were replaced with serum-free culture medium for another 12 h. The microglia culture supernatant was used to culture NSC34 neuronal cells for 12 h. For Transwell coculture system (Figure S2 B), the upper insert chamber was planted with BV-2 microglia cells and the lower six-well plate was used to implant NSC34 neuronal cells. In the LPS group, BV-2 microglia cells were treated with LPS at a concentration of 10 ng/mL for 6 h. In the last two groups, BV-2 microglia cells were pretreated with PK11195 (0.5 *μ*M) or Midazolam (15 *μ*M) for 1 h followed by LPS treatment for 6 h. Then, two kinds of cells were replaced with serum-free culture medium at the same time. The insert chamber was placed in combination with the six-well plate cocultivation for 12 h.

### 2.4. BV-2 Microglia and HT-22 Neuronal Cell Coculture System

The steps of the BV-2-HT-22 coculture system (Figure S3) were the same as those of BV-2-NSC34. The concentration of LPS was 1 *μg*/mL; the coculture time was 24 h in the BV-2-HT-22 coculture system.

### 2.5. Cell Viability Assay

Cell viability was measured by the Cell Counting Kit-8 (CCK-8) assay. Briefly, cells were seeded in 96-well culture plates and received the various treatments. After that, cells were incubated with 10 *μ*L CCK-8 reagent and finally, the light absorbance at 450 nm was measured using a microplate reader (BIO-RAD iMark).

### 2.6. Western Blot Analysis

Cultured cells were lysed in lysis buffer (Beijing Solarbio Science & Technology Co., Ltd.) on ice for 30 min, and the lysates were clarified by centrifugation at 4°C for 10 min. After quantitation of protein concentration using BCA Protein Assay Kit (CWBIO, Beijing, China), protein was heated at 100°C for 5 min. Each sample containing 20 *μ*g of protein was separated by 10% or 12% SDS-PAGE gels and transferred to PVDF membranes. The PVDF membranes were incubated with the primary antibodies at 4°C overnights. The primary antibodies are summarized in Table 1. The horseradish peroxidase- (HRP-) conjugated secondary antibodies (Goat anti-Rabbit, 1 : 7000 or Goat anti-Mouse, 1 : 7000; Proteintech, Wuhan, China) were used for 2 h at room temperature, and the expression of protein was evaluated by the enhanced chemiluminescence plus detection system (Tanon 4600, Shanghai, China).

### 2.7. RNA Isolation and Quantitative Real-Time PCR Assays

Total RNA was extracted with TRIzol reagent (Thermo Fisher Scientific, Shanghai, China) following the manufacturer's instructions. Isolated RNA was then reverse-transcribed into cDNA using the HiScript® III RT SuperMix (Vazyme, Nanjing, China) following the standard protocol. For real-time quantitative PCR analysis, the resultant cDNA products were amplified using a 2× ChamQ SYBR® qPCR Master Mix (Vazyme, Nanjing, China) in triplicate. The mRNA level was normalized to *β*-actin and expressed as fold change. The forward and reverse primer sequences are shown in Table 2.

### 2.8. Immunofluorescence (IF)

After treatment, the cells were treated with 4% paraformaldehyde for 30 min, then blocked in 5% goat blocking serum (Solarbio, Beijing, China) for 30 min at room temperature. Primary antibodies were incubated at 4°C overnight. Full details on primary antibodies used are provided in Table 3. Then, the cells were incubated with goat anti-rabbit IgG (1 : 500; Multi Sciences, Hangzhou, China) secondary antibody for 2 h in the dark at 37°C. Finally, fluorescence images were captured with a fluorescence microscope (Olympus, Tokyo, Japan), and the analysis of the fluorescence images was performed by ImageJ.

### 2.9. Transmission Electron Microscopy (TEM)

The BV-2 microglia cell precipitation was fixed in ice-cold 3% glutaraldehyde in phosphate buffer, pH 7.4, for 1 h, then postfixed in 1% OsO4. Following dehydration and embedding in EPON resin, ultrathin sections were cut and stained with uranyl acetate and lead citrate. Finally, the samples were imaged under the transmission electron microscope (HT 7700-SS, HITACHI, Japan).

### 2.10. Enzyme-Linked Immunosorbent Assay (ELISA)

IL-1*β* (Multi Sciences, Hangzhou, Zhejiang, China) and IL-18 (Multi Sciences, Hangzhou, Zhejiang, China) that the cells had secreted into the culture supernatant in the lower chamber in Transwell coculture system were measured by ELISA according to the manufacturer's instructions.

### 2.11. TUNEL

The apoptosis of neuronal cells was detected by CoraLite®594 TUNEL Assay Apoptosis Detection kit (Proteintech Group, Inc) according to the manufacturer's directions. The apoptotic cells were TUNEL positive that were labelled with red fluorescence under a fluorescence microscope. The ratio of TUNEL-positive cells to total neuronal cells indicates the apoptotic index.

### 2.12. Statistical Analyses

Data were presented as means ± SEM of three independent experiments. The data were analyzed with one-way ANOVA followed by Tukey's post hoc test for significance via SPSS 25.0. Asterisks indicate statistically significant difference between the compared groups: *P* < 0.05.

## 3. Results

### 3.1. TSPO Ligands Attenuated LPS-Induced High TSPO Expression in BV-2 Microglia Cells

**Figure 1:**
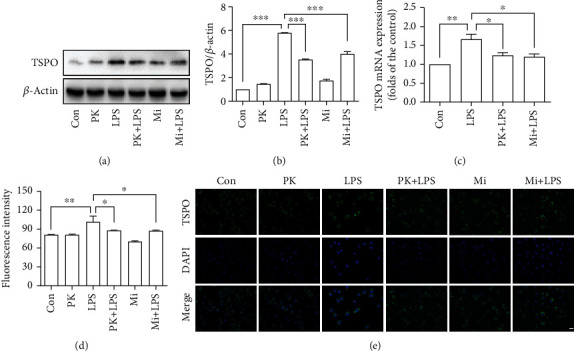
TSPO ligands attenuated LPS-induced high TSPO expression in BV-2 microglia cells. The expression of TSPO was quantified by Western blot (a, b), qRT-PCR (c), and immunofluorescence (d, e). Con: control; PK: PK11195; PK+LPS: PK11195+LPS; Mi: Midazolam; Mi+LPS: Midazolam+LPS. Scale bar, 20 *μ*m. ^∗^*P* < 0.05, ^∗∗^*P* < 0.01, and ^∗∗∗^*P* < 0.001.

We first investigated whether treatment with the LPS (1 *μ*g/mL) mediated the TSPO production in BV-2 microglia cells. As shown in Figure 1, the Western blot (Figures 1(a) and 1(b)), qRT-PCR (Figure 1(c)), and immunofluorescence (Figures 1(d) and 1(e)) results show that compared with the control group, TSPO protein level was significantly enhanced in the LPS group. Interestingly, compared with the LPS-treated cells, both the TSPO expressions in the PK11195+LPS group and the Midazolam+LPS group were reduced. These data indicate that TSPO ligands PK11195 and Midazolam attenuated LPS-induced high TSPO expression in BV-2 microglia cells.

### 3.2. TSPO Ligands Reversed the Inhibition of Mitophagy in LPS-Activated BV-2 Microglia Cells

**Figure 2:**
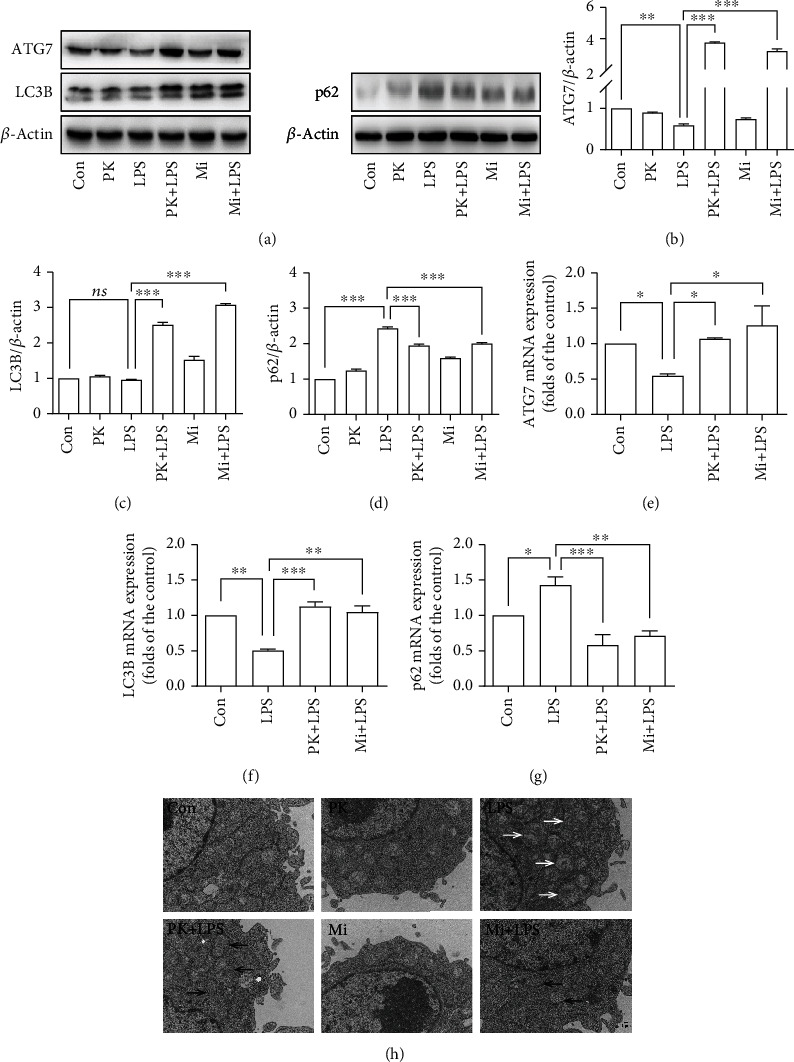
TSPO ligands reversed the inhibition of mitophagy in LPS-activated BV-2 microglia cells. (a–d) Representative images of ATG7, LC3B, p62, and *β*-actin expression in BV-2 microglia cells detected by Western blot. (e–g) Comparison of ATG7, LC3B, and p62 expression in BV-2 microglia cells by qRT-PCR. (h) TEM examined the ultrastructure of cells. Con: control; PK: PK11195; PK+LPS: PK11195+LPS; Mi: Midazolam; Mi+LPS: Midazolam+LPS. White solid arrow: swollen mitochondria; black solid arrow: autophagosomes. ^∗^*P* < 0.05, ^∗∗^*P* < 0.01, and ^∗∗∗^*P* < 0.001.

To explore the role of mitophagy in the regulation of microglia activation, we assessed the effects of LPS on the mitophagic process. The Western blot results showed that exposure to LPS (1 *μ*g/mL) reduces the expression of mitophagy-related proteins ATG7 (Figures 2(a) and 2(b)) and increases the expression of p62 (Figures 2(a) and 2(d)) compared with the control group. Immunofluorescence results (Figure S4) showed that the expression of ATG7 significantly reduced, and the expression of p62 increased in the LPS group compared with the control group. The qRT-PCR results that showed that the mRNA expression of ATG7 (Figure 2(e)) and LC3B (Figure 2(f)) was decreased, while the mRNA expression of p62 (Figure 2(g)) increased in the LPS group compared with the control group. These results suggested that exposure to LPS impaired mitophagy in BV-2 microglia cells. However, PK11195 or Midazolam pretreatment significantly reversed the inhibition of mitophagy by LPS, as demonstrated by the elevated expression of ATG7 (Figures 2(b) and 2(e)), LC3B (Figures 2(c) and 2(f)) and decreased expression of p62 (Figures 2(d) and 2(g)) in the PK11195+LPS or Midazolam+LPS group compared with the LPS group. Furthermore, TEM results (Figure 2(h)) showed that many swollen mitochondria were present in LPS-activated BV-2 microglia cells (white solid arrow). And there were more autophagosomes with swollen mitochondria (black solid arrow) observed in microglia cells pretreated with PK11195 or Midazolam followed by exposure to LPS. These results suggested that TSPO ligand PK11195 and Midazolam pretreatment can partially reverse LPS-induced inhibition of mitophagy in BV-2 microglia.

### 3.3. TSPO Ligands Inhibited Neuroinflammatory Reactions in LPS-Activated BV-2 Microglia Cells in the BV-2-NSC34 Transwell Coculture System

**Figure 3:**
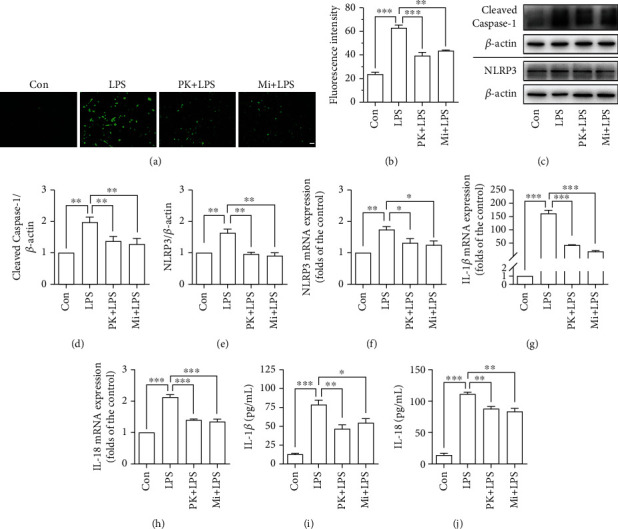
TSPO ligands inhibited neuroinflammatory reactions in LPS-activated BV-2 microglia cells in the BV-2-NSC34 Transwell coculture system. (a, b) Representative images showing the ROS production with DCFH-DA in BV-2 microglia cells. (c–e) Cleaved Caspase-1 and NLRP3 expression in BV-2 microglia cells based on Western blot analysis. (f–h) The mRNA expression of NLRP3, IL-1*β*, and IL-18 determined by qRT-PCR in BV-2 microglia cells. (i, j) The content of IL-1*β* and IL-18 in the BV-2-NSC34 Transwell coculture system determined by ELISA. Con: control; PK+LPS: PK11195+LPS; Mi+LPS: Midazolam+LPS. Scale bar, 20 *μ*m. ^∗^*P* < 0.05, ^∗∗^*P* < 0.01, and ^∗∗∗^*P* < 0.001.

Our previous study suggested that TSPO ligands can inhibit the activation of NLRP3 inflammasome in LPS-activated BV-2 microglia [21]. In the present study, we further investigated the activation of NLRP3 inflammasome in LPS-activated BV-2 microglia in BV-2-NSC34 Transwell coculture system. The results (Figures 3(a) and 3(b)) showed that the levels of ROS were significantly increased in BV-2 microglia cells in the BV-2-NSC34 Transwell coculture system. PK11195 or Midazolam pretreatment significantly inhibited the expression of ROS stimulated by LPS. Furthermore, the levels of Cleaved Caspase-1 and NLRP3 were measured by Western blot (Figures 3(c)–3(e)), qRT-PCR (Figure 3(f)), and immunofluorescence (Figure S5). The results showed that PK11195 or Midazolam pretreatment significantly inhibited the protein expression of Cleaved Caspase-1 (Figure 3(d)), NLRP3 (Figure 3(e)), and the mRNA expression of NLRP3 (Figure 3(f)) in BV-2 microglia cells. Moreover, the results showed that the mRNA levels of IL-1*β* (Figure 3(g)) and IL-18 (Figure 3(h)) were significantly increased, and pretreatment with PK11195 or Midazolam effectively inhibited the mRNA expression of IL-1*β* and IL-18. Then, in the BV-2-NSC34 Transwell coculture system, we found that the pretreatment with PK11195 or Midazolam could significantly reduce the content of IL-1*β* (Figure 3(i)) and IL-18 (Figure 3(j)) in the supernatant. Likewise, the pretreatment could significantly reduce the content of IL-1*β* and IL-18 in the lower chamber of the BV-2-HT-22 Transwell coculture system (Figure S6).

### 3.4. TSPO Ligands Prevented LPS-Activated Microglia-Induced Neurotoxicity in BV-2-NSC34 Coculture System

**Figure 4:**
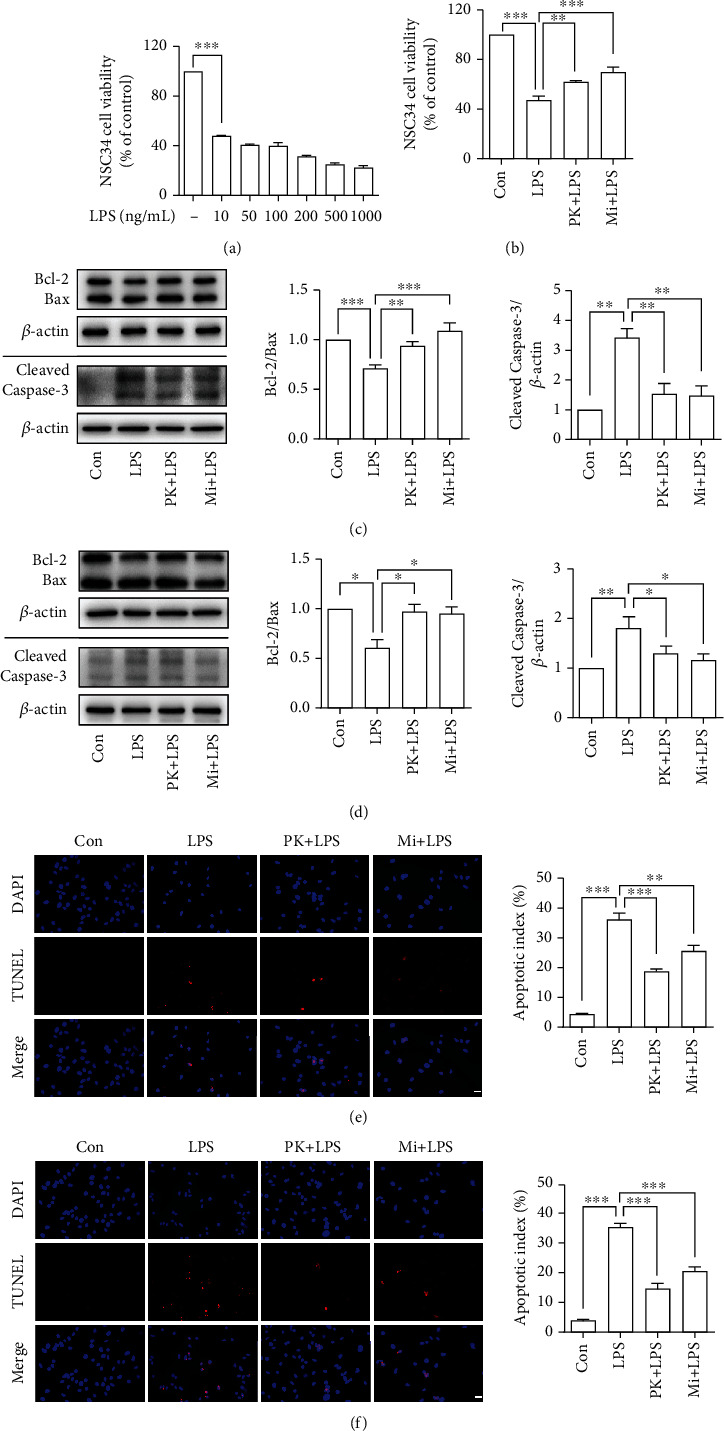
Anti-inflammatory effects of PK11195 and Midazolam on LPS-activated BV-2 cells protected NSC34 neuronal cells from cytotoxicity and provided neuroprotective effect against the NSC34 neuronal cell apoptosis. (a, b) The NSC34 neuronal cell viability detected by CCK-8. (c, d) The relative expression of apoptosis-related proteins Bcl-2, Bax, and Cleaved Caspase-3 in NSC34 neuronal cells under the CM (c) or Transwell (d) coculture system analyzed by Western blot. The NSC34 neuronal cell apoptosis determined by TUNEL staining in the CM (e) or Transwell (f) coculture system. Con: control; PK+LPS: PK11195+LPS; Mi+LPS: Midazolam+LPS. Scale bar, 20 *μ*m. ^∗^*P* < 0.05, ^∗∗^*P* < 0.01, and ^∗∗∗^*P* < 0.001.

We then investigated whether TSPO ligands could prevent neuroinflammation-mediated neuronal damage using the BV-2 microglia CM and the BV-2-NSC34 Transwell coculture system. First, we use different concentrations of LPS to stimulate microglia and used the media to process NSC34 neuronal cells, so as to select the appropriate LPS stimulation concentration. Based on the results shown in Figure 4(a), we selected 10 ng/mL LPS to stimulate the BV-2 in the CM coculture system or BV-2-NSC34 Transwell coculture system. As expected, pretreatment with PK11195 or Midazolam prevented NSC34 neuronal cells against activated microglia-induced cytotoxicity and increased the neuronal cell viability (Figure 4(b)) in the CM coculture system. Furthermore, we used Western blot to detect the expression of apoptosis-related proteins Bcl-2 (B-cell lymphoma-2), Bax (Bcl2-Associated X), and Cleaved Caspase-3 in NSC34 neuronal cells (Figures 4(c) and 4(d)). The results of Bcl-2/Bax indicated that PK11195 and Midazolam pretreatment could protect against NSC34 neuronal cells from apoptosis, as evidenced by increasing in the ratio of Bcl-2/Bax compared with the LPS-treated group in the CM (Figure 4(c)) or Transwell (Figure 4(d)) coculture system. In addition, compared with the control group, LPS stimulation significantly increased Cleaved Caspase-3 activity in NSC34 neuronal cells in the CM (Figure 4(c)) and Transwell (Figure 4(d)) coculture system, whereas PK11195 and Midazolam incubation resulted in reduction of Cleaved Caspase-3 expression. Meanwhile, TUNEL staining was used to monitor the apoptosis of NSC34 neuronal cells. It was found that LPS stimulation induced the increase of apoptotic cells (Figures 4(e) and 4(f)). These changes were attenuated by pretreatment with PK11195 and Midazolam evidenced by a decreased number of apoptotic cells in the CM (Figure 4(e)) or Transwell (Figure 4(f)) coculture system.

### 3.5. TSPO Ligands Prevented LPS-Activated Microglia-Mediated Neurotoxicity in BV-2-HT-22 Coculture System

**Figure 5:**
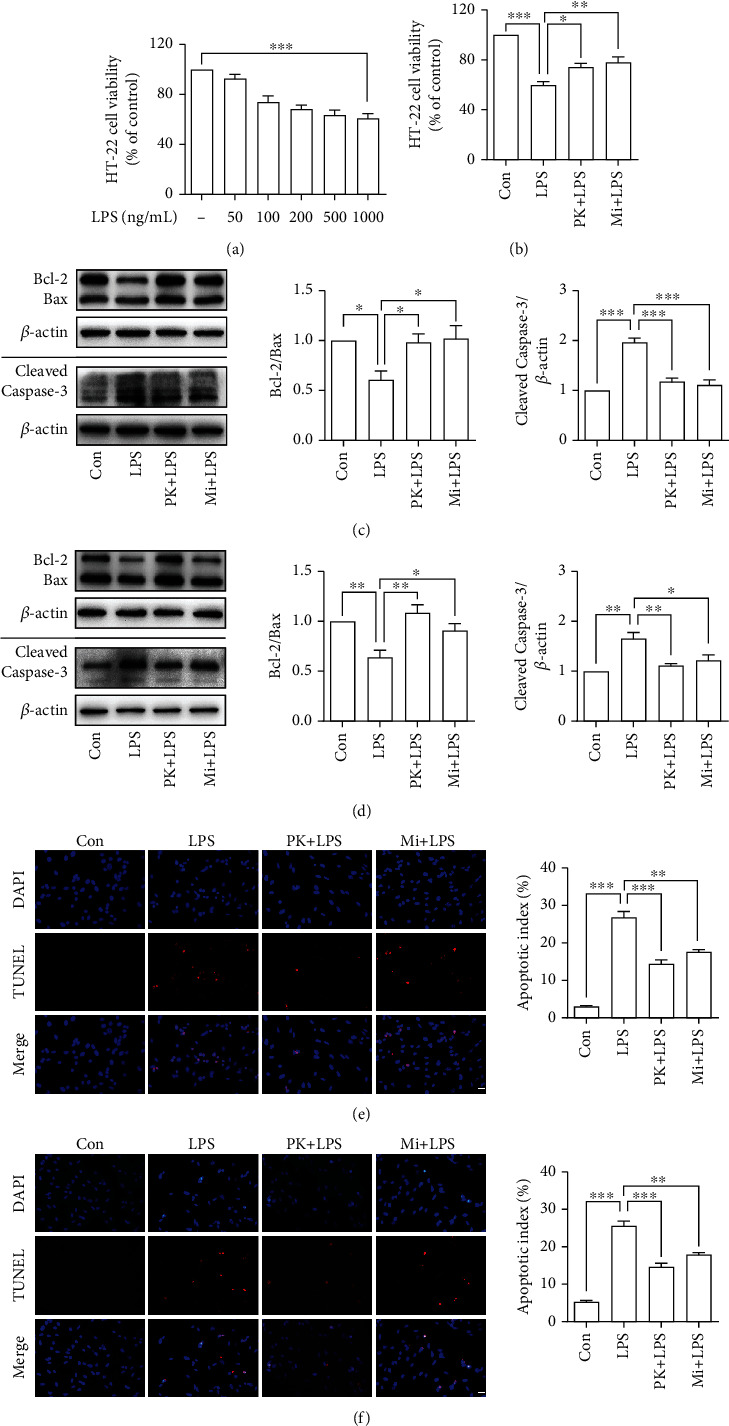
Anti-inflammatory effects of PK11195 and Midazolam on LPS-activated BV-2 cells protected HT-22 neuronal cells from cytotoxicity and provided neuroprotective effect against the HT-22 neuronal cell apoptosis. (a, b) The HT-22 neuronal cell viability detected by CCK-8. (c, d) The relative expression of apoptosis-related protein Bcl-2, Bax, and Cleaved Caspase-3 in HT-22 neuronal cells under the CM (c) or Transwell (d) coculture system analyzed by Western blot. The HT-22 neuronal cell apoptosis determined by TUNEL staining in the CM (e) or Transwell (f) coculture system. Con: control; PK+LPS: PK11195+LPS; Mi+LPS: Midazolam+LPS. Scale bar, 20 *μ*m. ^∗^*P* < 0.05, ^∗∗^*P* < 0.01, and ^∗∗∗^*P* < 0.001.

Similarly, as shown in Figure 5(a), 1 *μ*g/mL LPS was used to stimulate microglia in the CM or BV-2-HT-22 Transwell coculture system. As illustrated in Figure 5(b), pretreatment with PK11195 and Midazolam prevented HT-22 neuronal cells against activated microglia-induced cytotoxicity in the CM coculture system. It was found that PK11195 and Midazolam pretreatment could increase the ratio of Bcl-2/Bax compared with the LPS group in the CM (Figure 5(c)) or Transwell (Figure 5(d)) coculture system. In addition, PK11195 and Midazolam incubation resulted in reduction of Cleaved Caspase-3 expression in the CM (Figure 5(c)) or Transwell (Figure 5(d)) coculture system. As illustrated in Figures 5(e) and 5(f), TUNEL staining has shown that LPS stimulation induced the increase of apoptotic cells. However, these changes were attenuated by pretreatment with PK11195 and Midazolam evidenced by a decreased number of apoptotic cells.

## 4. Discussion

Our current results showed that TSPO ligands PK11195 and Midazolam played an anti-inflammatory role by improving mitophagy to inhibit NLRP3 inflammasome activation in BV-2 microglia cells. In the microglia-neuron coculture system, TSPO ligand pretreatment reduced the damage of microglia inflammatory response to neurons, thus playing a neuroprotective role.

**Figure 6:**
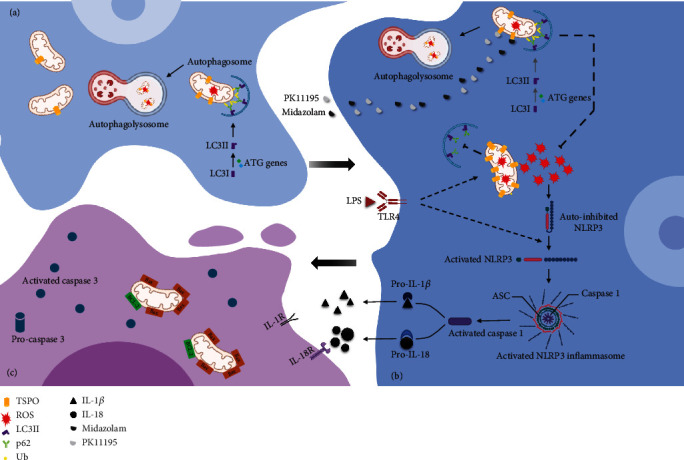
In resting microglia (a), normal mitophagy maintains mitochondrial homeostasis. In LPS-activated microglia (b), the expression of TSPO in the outer membrane of mitochondria is increased, which hinders the recognition and clearance of damaged mitochondria by autophagosomes. ROS produced by damaged mitochondria accumulate and activate NLRP3 inflammasome, causing the activation of caspase-1. Activated caspase-1 promotes extracellular secretion of IL-1*β* and IL-18. Under the stimulation of inflammatory cytokines, neuronal cells undergo apoptosis (c). Application of TSPO ligands PK11195 and Midazolam improved mitophagy and inhibited the accumulation of ROS to block the inflammatory response in LPS-activated microglia, to protect neuronal cells.

Under normal circumstances, TSPO expressed at low level in the CNS and high level of TPSO in the brain especially in activated microglia suggests a significant injury and inflammation [13, 22]. *In vivo* human imaging studies have shown that TSPO levels in activated glial cells are increased, especially in neurodegenerative diseases [17]. In the present study, BV-2 microglia cells were used in the research. After activation of BV-2 microglia cells by LPS, the expression of TSPO was significantly increased. Meanwhile, in the BV-2-NSC34 Transwell coculture system, stimulation with LPS also caused high TSPO expression in microglia cells (Figure S7). Researches over the last few decades have emphasized on the interaction of TSPO with its ligands; the most common one is PK11195 [12]. TSPO ligands have shown promising therapeutic prospects in various studies [14, 15]. Previous studies in our laboratory showed that PK11195 and Midazolam have anti-inflammatory action *in vitro* [21]. In addition, we also found that PK11195 could regulate the process of mitophagy [23]. Mitophagy, selective autophagy of mitochondria, plays an important role in the development and maintenance of the CNS [23–25] which can help cells adapt to the living environment [26]. When mitochondria are damaged by external stimuli, the oxidative stress system is disrupted. Mitophagy can clear the damaged mitochondria to control their own quality and thus act as an anti-inflammatory and neuroprotective agent [27, 28]. Therefore, we further examined the regulatory effect of PK11195 and Midazolam on mitophagy in microglia cells *in vitro*. In the current study, Western blot, qRT-PCR, and immunofluorescence results showed that the expression of mitophagy-related proteins ATG7, p62, and LC3B in LPS-activated BV-2 microglia cells was disorganized, as evidenced by a decrease in ATG7 and LC3B and an increase in p62. The current mechanistic model of mitophagy requires the PTEN-induced kinase 1 (PINK1) to accumulate on the outer membranes of damaged mitochondria, where it initiates the recruitment of and Parkin RBR E3 ubiquitin-protein ligase (PARK2). PARK2 ubiquitinates outer mitochondrial membrane proteins and recruits p62 (Figures 6(a) and 6(b)). p62 further recruits LC3B, which promotes autophagic degradation of ubiquitination-tagged damaged mitochondria [2, 29]. The decrease of ATG7 and LC3B was not conducive to the binding of damaged mitochondria and autophagosomes. The increase of p62 indicates that the further degradation process of autophagosomes was hindered [23]. Therefore, the results of ATG7, LC3B, and p62 in BV-2 microglia supported the impairment of mitophagy, which could be partially restored after pretreatment with TSPO ligands PK11195 and Midazolam. Furthermore, TEM analysis showed that LPS-induced autophagy appeared to occur in the early stages of autophagy formation, because a lot of autophagic vacuoles were observed. This helped to show that further recognition and clearance of damaged mitochondria by autophagosome were hindered. In contrast, after pretreatment with PK11195 or Midazolam before exposure to LPS, more mature autophagosomes and advanced autophagic lysosome structures were observed, indicating that the damaged mitochondria are further removed and recycled. Overall, pretreatment of TSPO ligands contributes to the clearance of damaged mitochondria by increasing mitochondrial autophagy.

The dysregulation of the mitophagy pathway could lead to the accumulation of damaged mitochondria, resulting in increased oxidative stress [20]. In a previous study by our group, we found that ROS were significantly elevated in LPS-activated microglia [21]. And in the current study, we found the high levels of ROS in LPS-activated BV-2 microglia in the BV-2-NSC34 coculture system. The production of ROS promotes activation of NLRP3 which can further inhibit the clearance of mitochondria, thus creating a vicious cycle [19]. It has been reported that the expression of NLRP3 in the brain of elderly rats is significantly higher than that of young rats, and the activation of NLRP3 is significantly increased in neurodegenerative diseases [30, 31]. In our study, PK11195 and Midazolam pretreatment can inhibit the activation of NLRP3 by reducing the production of ROS. The activation of NLRP3 inflammasome could induce secretion of IL-18 and IL-1*β* [32]. Therefore, the content of IL-18 and IL-1*β* in the Transwell coculture system was investigated and it was found that the content of IL-1*β* and IL-18 increased in the LPS group. These inflammatory cytokines cause vascular endothelial cell damage around activated microglia and disrupt the tight junctions of astrocytes, thereby damaging the blood-brain barrier. Further accumulation of activated microglia exacerbates the neuroinflammatory response [1]. Fortunately, PK11195 as well as Midazolam could reduce the content of IL-1*β* and IL-18 secreted by LPS-activated microglia, thus blocking the neuronal damage by neuroinflammation [33].

In the present study, two types of coculture systems, i.e., CM coculture system and Transwell coculture system [34–37], were used to verify the neuroprotective effects of PK11195 and Midazolam. In addition, two different neuronal cell lines were used to simulate the motor neurons and the hippocampal neurons in this study. Among them, NSC34 cells were often used to the study of amyotrophic lateral sclerosis [38] and HT-22 hippocampal neuron cells were often used to study cognitive impairment [35] *in vitro*. The data showed that PK11195 and Midazolam preconditioning alleviated the decrease in neuronal activity induced by LPS-activated microglia, including NSC34 and HT-22 neuronal cells in the CM coculture system. One of the main features of neurodegenerative diseases is the progressive loss of neuronal cells, and the main mode of loss includes the initiation of apoptotic programs following various stimuli to neurons [26]. These stimuli include foreign inflammatory cytokines, mechanical damage, or intrinsic senescent organelles that cannot be effectively removed. These factors eventually cause activation of caspase-3 in neuronal cells and thus mediate progressive apoptosis of neurons. So, we detected the apoptosis of NSC34 and HT-22 neuronal cells under two coculture systems by detecting apoptosis-related proteins Bcl-2, Bax, and Cleaved Caspase-3 and apoptotic cell staining. Our data indicated that PK11195 and Midazolam pretreatment could reduce the apoptosis of NSC34 and HT-22 neuronal cells, both in the CM coculture system and in the Transwell coculture system. In this study, the decrease in neuronal viability and apoptosis of neuron may be due to inflammatory cytokines secreted by abnormally activated microglia or other undetected substances, such as fragmented mitochondria [39]. From the results of this research, it is known that PK11195 and Midazolam may protect neurons from abnormally activated microglia damage by inhibiting the inflammatory response (Figure 6). Whether neurons are protected by other means, such as inhibiting mitochondrial release from microglia, is something that needs to be further explored.

## 5. Conclusions

Our current study demonstrated that TSPO ligands PK11195 and Midazolam showed neuroprotective effects by reducing the inflammatory response of LPS-activated BV-2 microglia, which may be related to the enhancement of mitophagy and the inhibition of NLRP3 inflammasome.

## Figures and Tables

**Table 1:** The primary antibodies used in Western blot.

Antibody	Company	Catalogue number	Source	Dilution
*β*-Actin	Beyotime	AA128	Mouse	1 : 1000
PBR	Abcam	ab109497	Rabbit	1 : 10000
ATG7	Abcam	ab133528	Rabbit	1 : 10000
LC3B	Abcam	ab192890	Rabbit	1 : 2000
p62	Abcam	ab109012	Rabbit	1 : 10000
NLRP3	Abcam	ab263899	Rabbit	1 : 1000
Cleaved Caspase-1	Affinity	AF4022	Rabbit	1 : 1000
Bcl2	Affinity	AF6139	Rabbit	1 : 1000
Bax	Affinity	AF0120	Rabbit	1 : 1000
Cleaved Caspase-3	Affinity	AF7022	Rabbit	1 : 1000

**Table 2:** Primer sequences for qRT-PCR.

Gene	Forward (5′-3′)	Reverse (3′-5′)
*β*-Actin	CTAAGGCCAACCGTGAAAAG	ACCAGAGGCATACAGGGACA
TSPO	GCTGTGGATCTTTCCAGAACA	ATGCCAAGAGGGTTTCTGC
ATG7	ATGCCAGGACACCCTGTGAACTTC	ACATCATTGCAGAAGTAGCAGCCA
LC3B	GAAGACCTTCAAACAGCGCC	CTTGGTCTTGTCCAGGACGG
p62	ATGGACATGGGGAGCTICAA	GTGCTCTCTGTATGCTCCCT
NLRP3	CCTGGGGGACTTTGGAATCAG	GATCCTGACAACACGCGGA
IL-1*β*	GCCCATCCTCTGTGACTCAT	AGGCCACAGGTATTTTGTCG
IL-18	GCCTGTGTTCGAGGATATGACT	CCTTCACAGAGAGAGGGTCACAG

**Table 3:** The primary antibodies used in immunofluorescence.

Antibody	Company	Catalogue number	Source	Dilution
PBR	Abcam	ab109497	Rabbit	1 : 1000
ATG7	Abcam	ab133528	Rabbit	1 : 1000
LC3B	Abcam	ab192890	Rabbit	1 : 1000
p62	Abcam	ab109012	Rabbit	1 : 1000
NLRP3	Abcam	ab263899	Rabbit	1 : 200
Cleaved Caspase-1	Affinity	AF4022	Rabbit	1 : 200

## Data Availability

The data used to support the findings of this study are included within the article.

## Supplementary Materials

Supplementary Materials Figure S1: BV-2 microglia treatment. Figure S2: BV-2-NSC34 coculture system. Figure S3: BV-2-HT-22 coculture system. Figure S4: ATG7, LC3B, and p62 expression with IF in BV-2 microglia cells. Figure S5: Cleaved Caspase-1 and NLRP3 expression with IF in BV-2 microglia cells in BV-2-NSC34 Transwell coculture system. Figure S6: the content of IL-1*β* and IL-18 in BV-2-HT-22 Transwell coculture system. Figure S7: the expression of TSPO in BV-2 microglia cells in BV-2-NSC34 Transwell coculture system.
