# Supplementary material for: TSPO Ligands Protect against Neuronal Damage Mediated by LPS-Induced BV-2 Microglia Activation
Source: Oxid Med Cell Longev. 2022 Mar 30;2022:5896699. doi: 10.1155/2022/5896699 (PMC8986436; doi:10.1155/2022/5896699)
Supplement: Supplementary Materials — Figure S1: BV-2 microglia treatment. Figure S2: BV-2-NSC34 coculture system. Figure S3: BV-2-HT-22 coculture system. Figure S4: ATG7, LC3B, and p62 expression with IF in BV-2 microglia cells. Figure S5: Cleaved Caspase-1 and NLRP3 expression with IF in BV-2 microglia cells in BV-2-NSC34 Transwell coculture system. Figure S6: the content of IL-1β and IL-18 in BV-2-HT-22 Transwell coculture system. Figure S7: the expression of TSPO in BV-2 microglia cells in BV-2-NSC34 Transwell coculture system. [file 5896699.f1.docx]

Supplementary Material

#
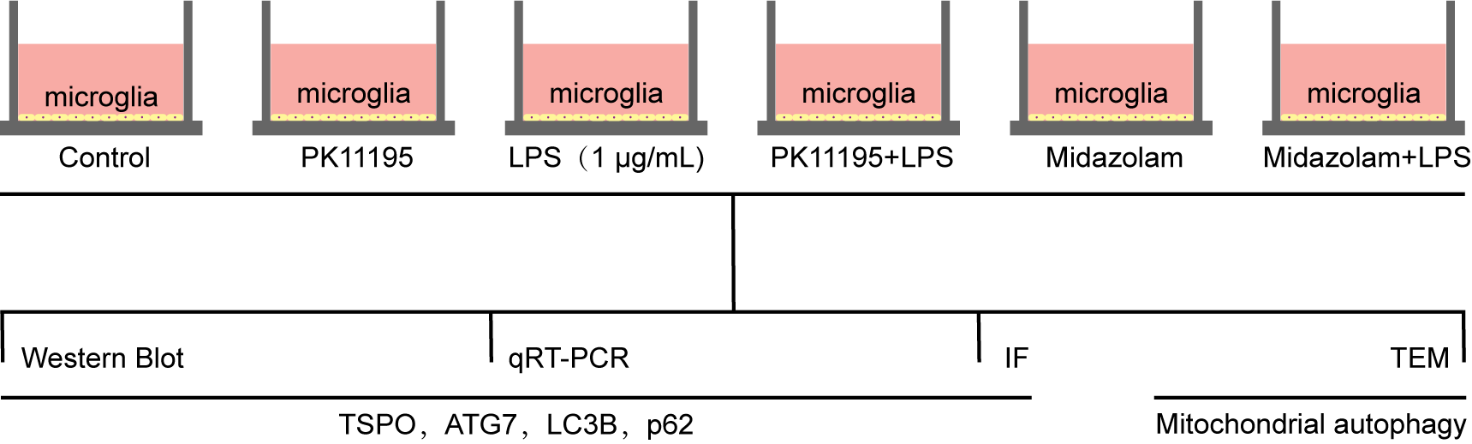


# Figure S1: BV-2 microglia treatment. The BV-2 microglia cells were randomly divided into six groups including Control group, PK11195 group, LPS group, PK11195+LPS group, Midazolam group, and Midazolam+LPS group. In both PK11195+LPS group and Midazolam+LPS group, cells were pre-treated with PK11195 or Midazolam for 1 h before treating with LPS for 6 h.

#
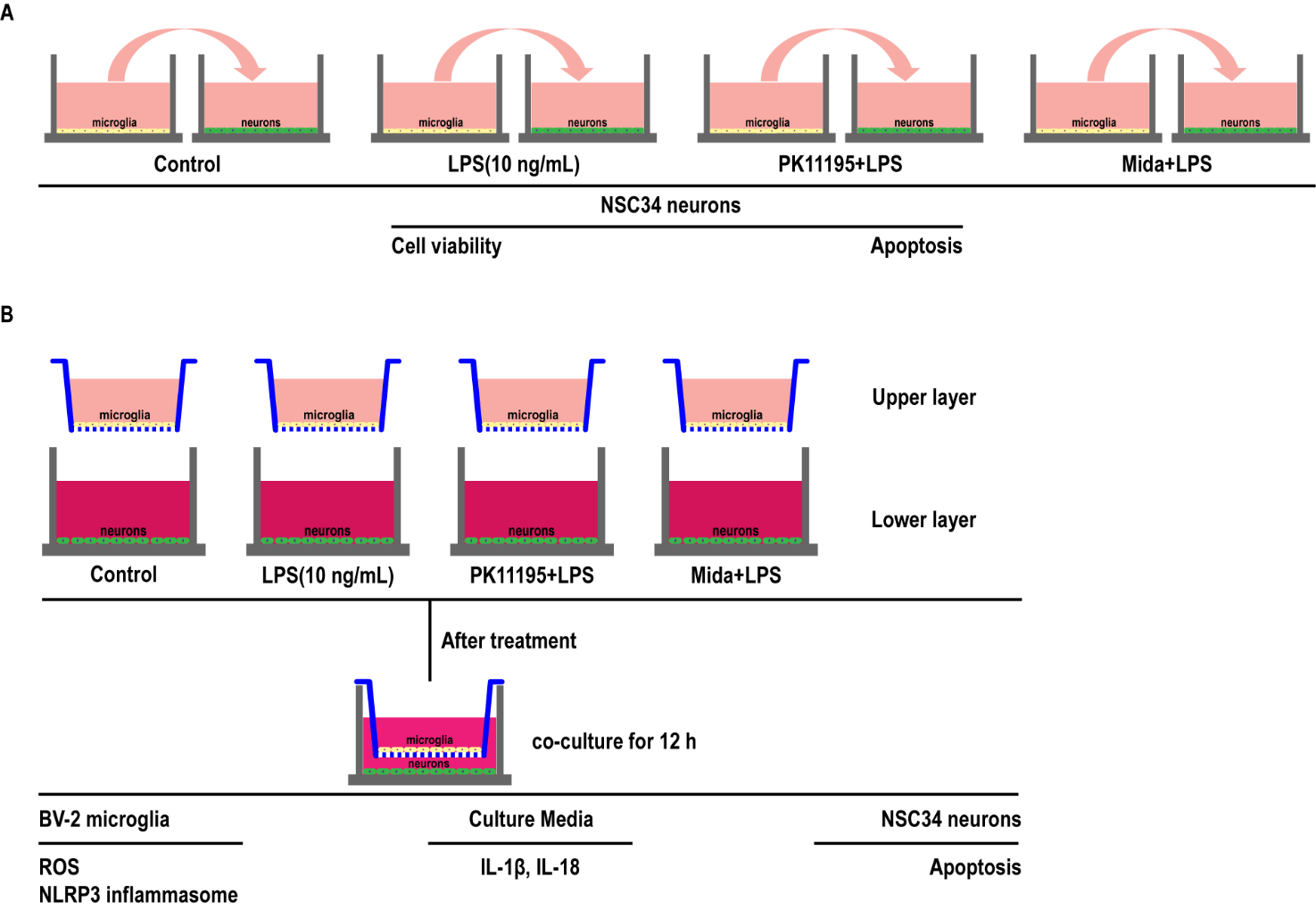


# Figure S2: BV-2-NSC34 co-culture system. (A) conditioned medium co-culture system. (B) Transwell co-culture system. Mida+LPS: Midazolam+LPS.

#
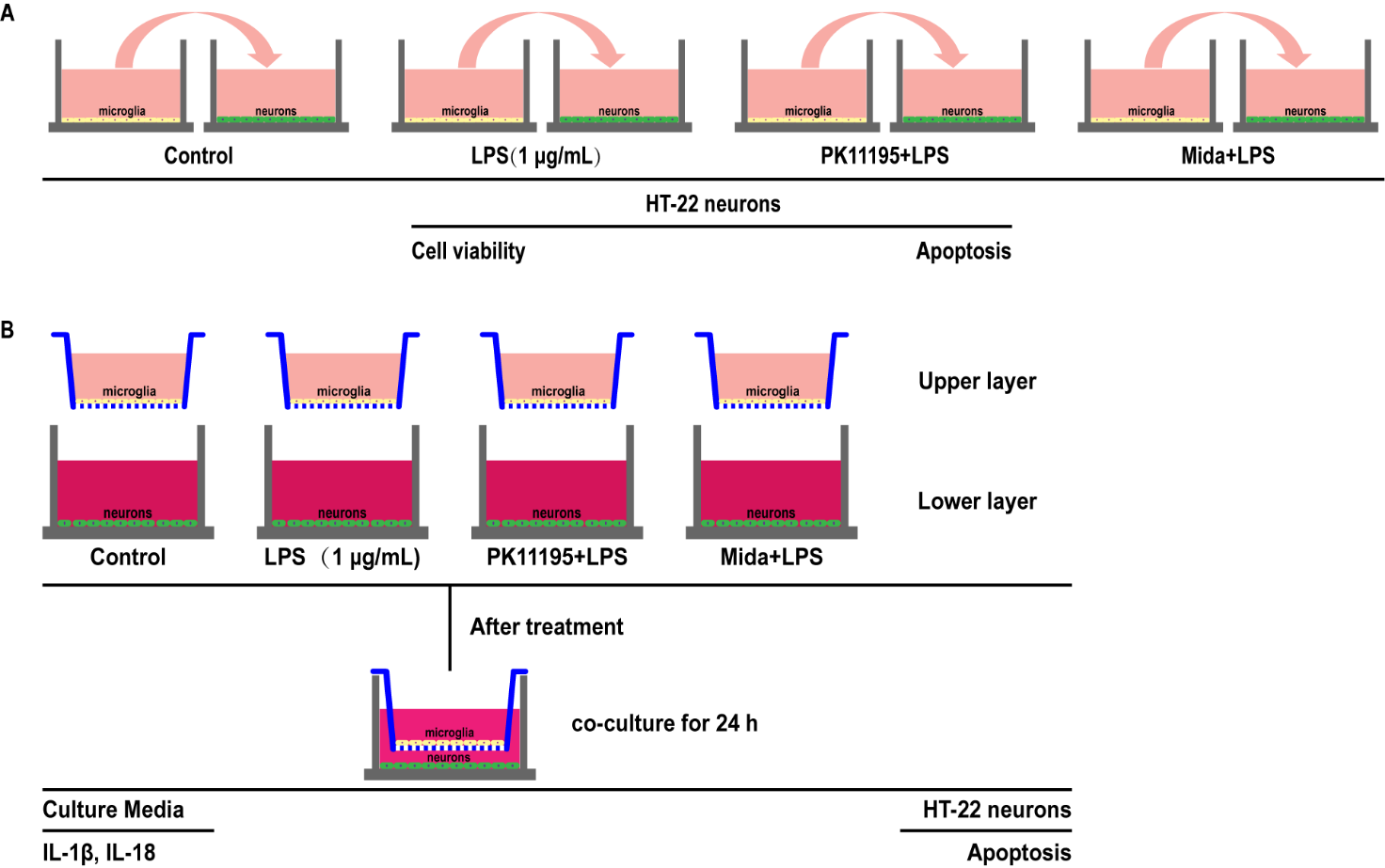


# Figure S3: BV-2-HT-22 co-culture system. (A) conditioned medium co-culture system. (B) Transwell co-culture system. Mida+LPS: Midazolam+LPS.


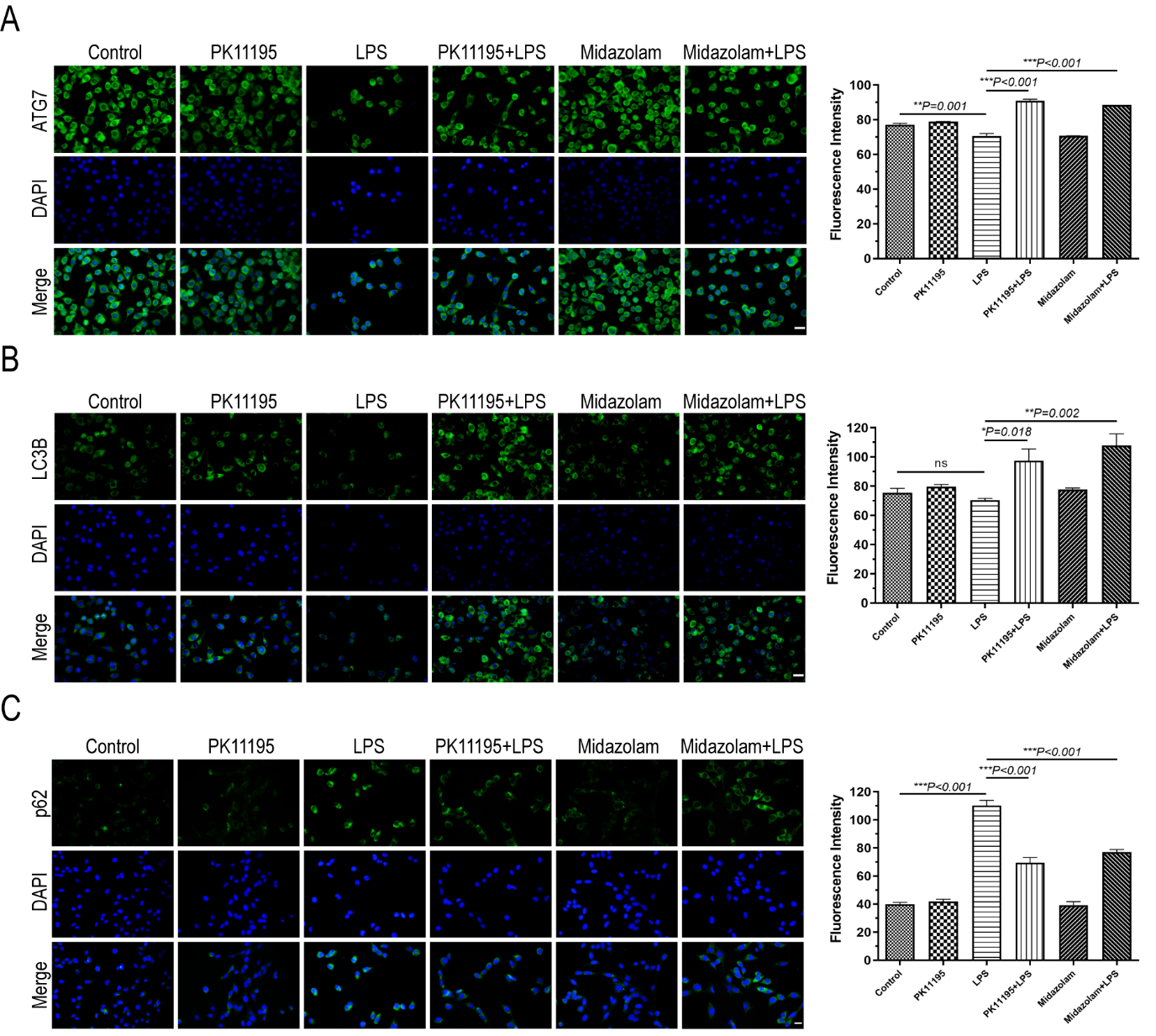


**Figure S4**: Representative images showing ATG7 **(A)**, LC3B **(B)** and p62 **(C)** expression with immunofluorescence in BV-2 microglia cells. Scale bar, 20 µm. ^*^*P* < 0.05, ^**^*P*<0.01, ^***^*P*<0.001


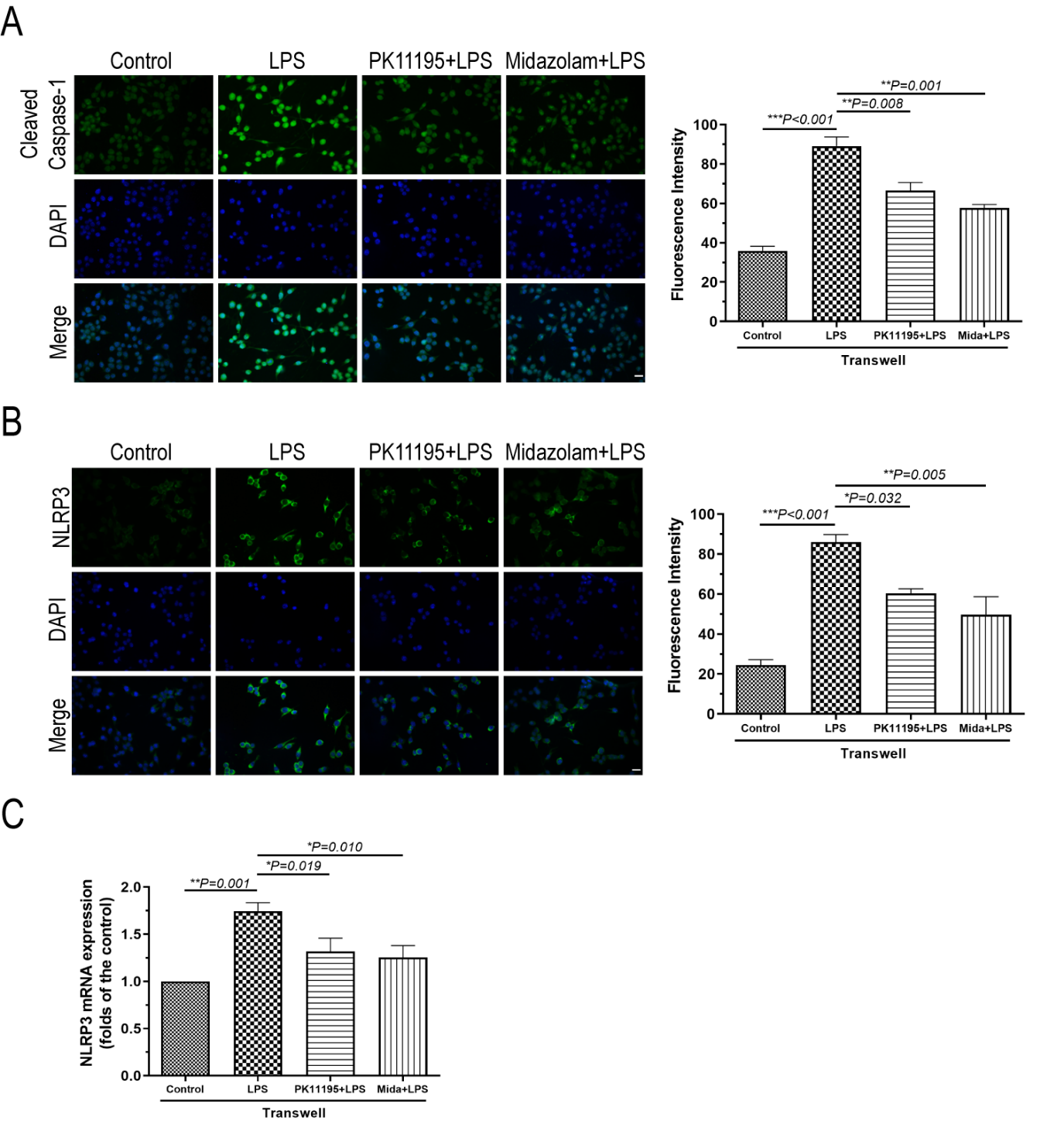


**Figure S5**: Representative images showing Cleaved Caspase-1 **(A)** and NLRP3 **(B)**expression with immunofluorescence in BV-2 microglia cells in BV-2-NSC34 Transwell co-culture system. Mida+LPS: Midazolam+LPS. Scale bar, 20 µm. ^*^*P* < 0.05, ^**^*P*<0.01, ^***^*P*<0.001


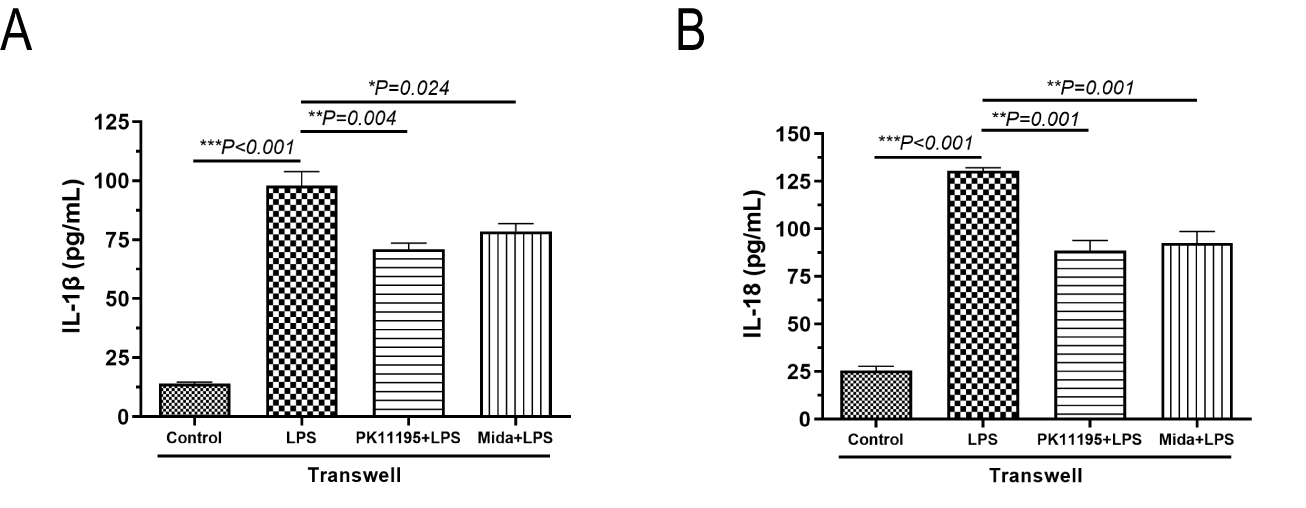


**Figure S6**: The content of IL-1β **(A)** and IL-18 **(B)** in BV-2-HT-22 Transwell co-culture system determined by ELISA. Mida+LPS: Midazolam+LPS. ^*^*P* < 0.05, ^**^*P*<0.01, ^***^*P*<0.001


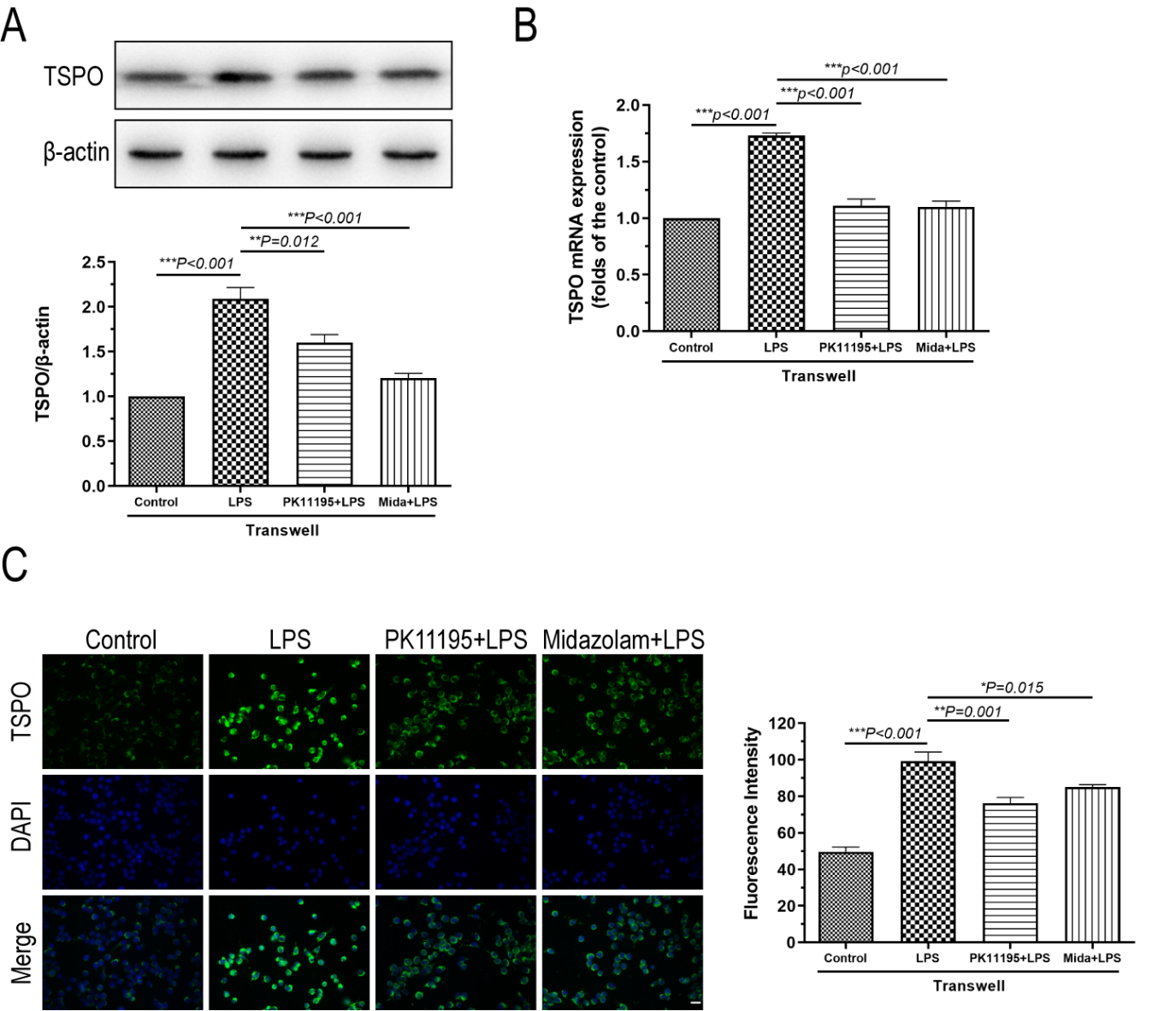


**Figure S7**: The expression of TSPO by the Western blot **(A)**, qRT-PCR **(B)** and immunofluorescence **(C)** in BV-2 microglia cells in BV-2-NSC34 Transwell co-culture system. Mida+LPS: Midazolam+LPS. Scale bar, 20 µm. ^*^*P* < 0.05, ^**^*P*<0.01, ^***^*P*<0.001
